# Supplementary material for: The anatomy of transcriptionally active chromatin loops in Drosophila primary spermatocytes using super-resolution microscopy
Source: PLoS Genet. 2023 Mar 3;19(3):e1010654. doi: 10.1371/journal.pgen.1010654 (PMC10016678; doi:10.1371/journal.pgen.1010654)
Supplement: S1 Information — (DOCX) [file pgen.1010654.s001.docx]

**The anatomy of transcriptionally active chromatin loops in *Drosophila* primary spermatocytes using super-resolution microscopy: supplementary data**

**1.*Camera alignment and denoise procedure***

***Supplementary Figure 1 (available separately as S1 Fig).*** *(A) Demonstration of the outcome of manual alignments of the camera using a 100 nm bead. The super-resolution SMLM analysis process is run on the bead, producing a few localisations at the central position of the bead. Then, the misalignment between the camera is corrected, and the analysis re-tried. This process is repeated until the two resulting localisations in both cameras are directly on top of each other, showing that the cameras are aligned. Scale bar is 100 nm. (B) Comparison of raw and N2V3D denoised images. Scale bars, 3 µm.*

For the Z-stack STORM images in Fig 2D and E, and the STORM image used for Figure 8B and 8D FIJI plugin Template Matching was used to re-align the slices in the stack after thermal drift between the image acquisitions, and a misalignment of the two cameras respectively using co-labelled structures on the cell surface as a reference point.

***2. Transcription Inhibition***

***
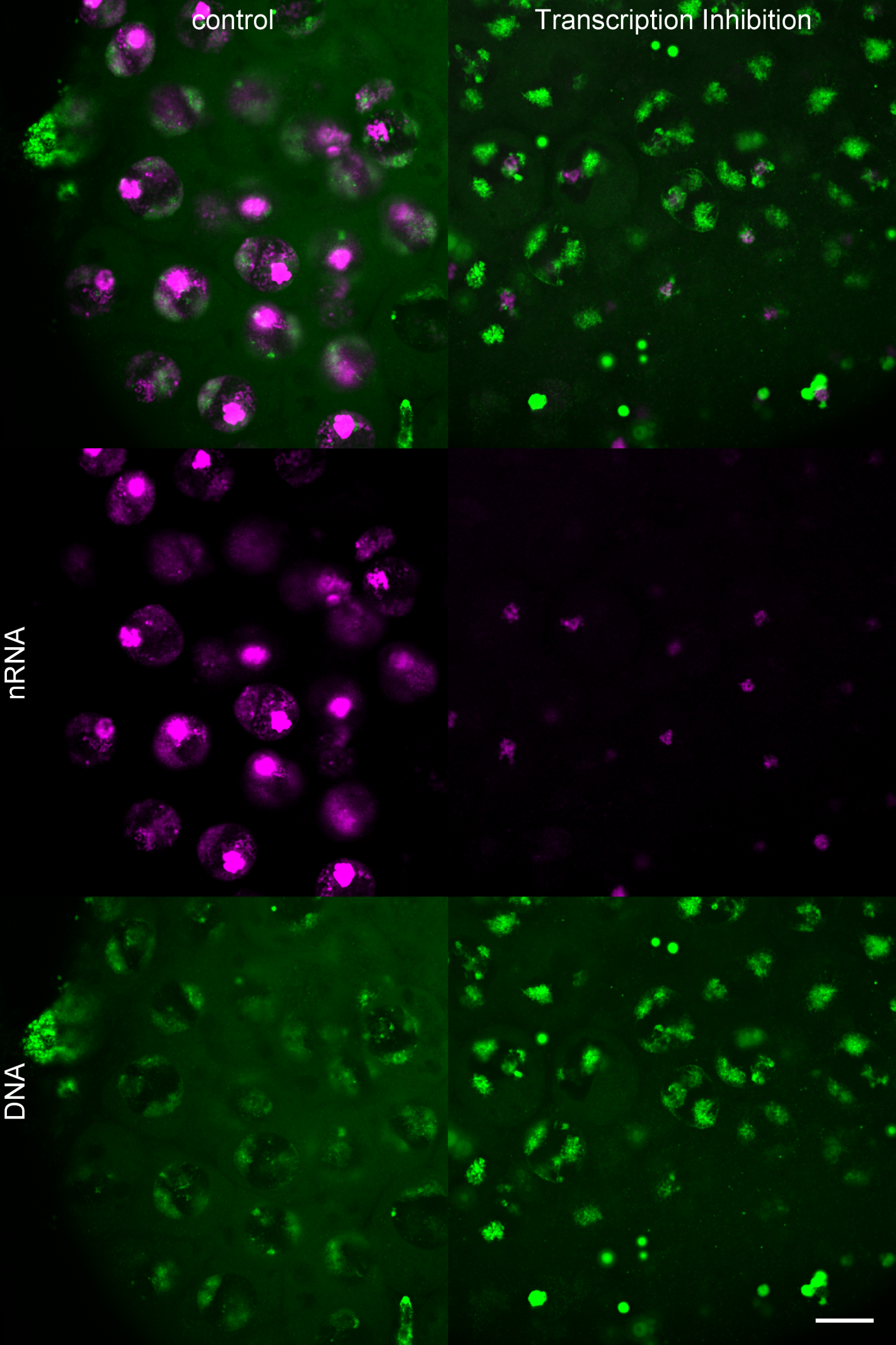
***

***Supplementary Figure 2.*** *Validation of transcription inhibition. Single Airyscan confocal slices of whole-mount testes after control (left panels) or transcription inhibition treatment (right panels) which show a strong decrease in EU incorporation into nascent RNA during a 45 min period (middle row). Top row: merge of stained nascent RNA (nRNA; middle row) and DAPI staining (bottom row). Single slices of 16 bit tiffs were combined in Fiji so that the display settings apply to both conditions equally, arranged in Fiji and saved as RGB Tiff. Display settings in the nRNA channel were chosen so that nRNA is apparent along nucleoplasmic Y loops in the control, thus the nucleolus appears saturated (middle row, left panel). In the treated testes considerable amounts of RNA can only be detected in the nucleolus and are most likely generated by RPol I (middle row, right panel). Scale bar, 10 µm.*

The transcription inhibition procedure is described in the main Materials and Methods section. Confocal imaging was performed on a Zeiss LSM 900 with Airyscan 2 with optimal Airyscan settings using a Plan-Apochromat 63x NA1.4 Oil DIC M27 objective, 638 and 405 nm excitation lasers, a pixel time of 0.96 µs, 4x line averaging with a detector gain of 850 V. Image size was 77.16 x 77.16 µm, voxel size 35 x 35 x 150 nm with 16 Bit. Airyscan SuperResolution processing was set at 7.6 for the Alexa647 channel (EU) and at 6.9 for DAPI. The stack depth was about 25 µm.

***Cluster Quantification***


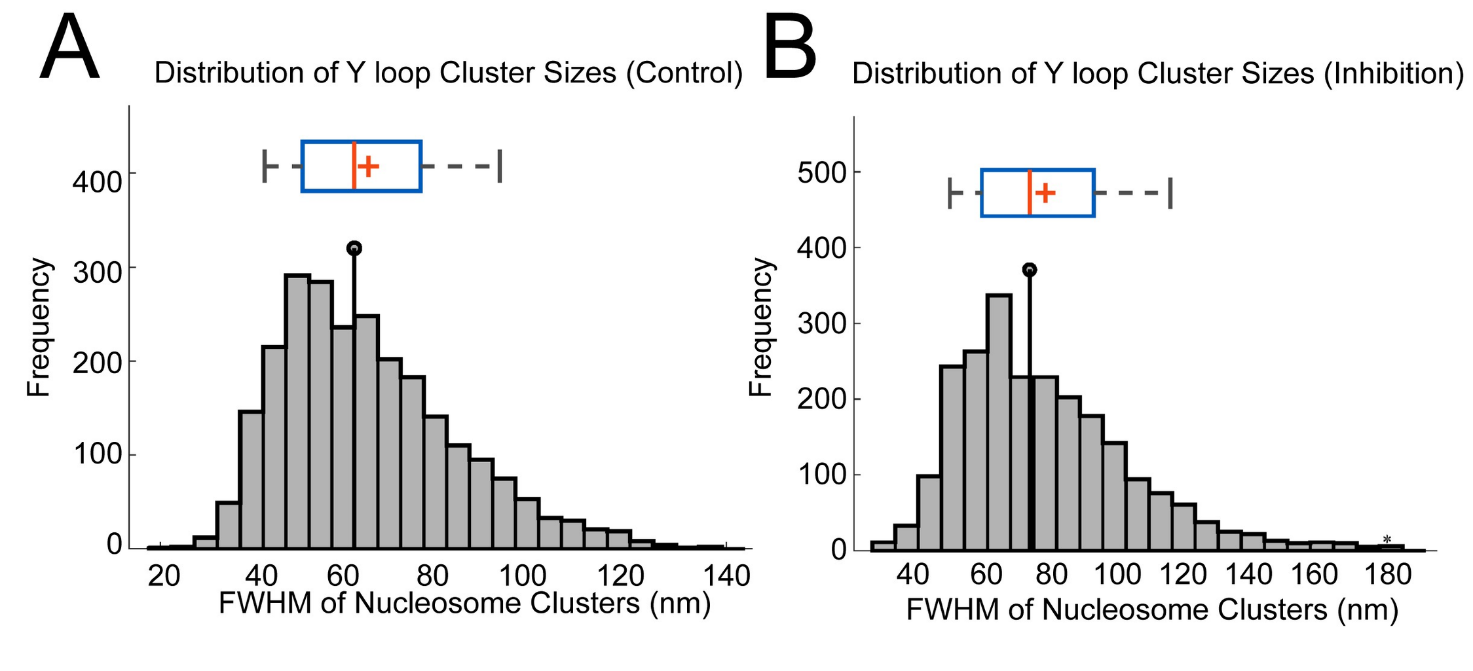


***Supplementary Figure 3.*** *(A) The total distribution of FWHMs of the control nucleosome clusters along the Y loops displayed with a histogram, and box and whiskers plot (the box indicates the inter-quartile range and the whiskers show the 9% and 91% bounds). The median FWHM was 64 nm, indicated with a lollipop on the histogram, and a line on the box and whiskers plot. The mean was 53 nm, indicated by a ‘+’ on the box and whiskers plot. Total cluster n = 2461, from 9 cells. (B) Histogram of the FWHMs of the transcription inhibition nucleosome clusters along the Y loops, the median is 73 nm. The box and whisker plots are the same as in A. Total cluster n = 2336, from 9 cells.*

The median FWHM of the larval testes control Y loop chromatin clusters was 61 nm, which was larger than the median FWHM shown in the adult testes primary spermatocytes (52 nm). It is not known why this is the case, although there may have been a slight effect on the Y loop phenotype caused by culturing in the medium for 20 hours, or perhaps some effect of the added DMSO to match the added DMSO in the stock solutions of α-amanitin and triptolide. Visually, there does not appear to be any effect on the regular Y loop clusters. The median FWHM for the transcription inhibition Y loop chromatin clusters was 73 nm, representing an approximate 20% increase in size compared to the control Y loops. Both a two-sample Kolmogorov-Smirnov statistical test and Students T test rejected the null hypothesis, demonstrating that the two distributions are significantly different. However, as the ROIs are cropped indiscriminately along the Y loop lengths, this is likely due to the more common presence of larger clusters along the Y loops formed either by twisting, or collapsing of the Y loops into one another (potentially through loss of bulk RNA mass), rather than an actual enlargement of the regular chromatin clusters that make up the Y loop fibres. To test this hypothesis, a smaller sub-section of Y loop chromatin clusters in transcription inhibited cells that were clearly separated from other fibres, and showed no collapsing or major twisting were quantified.


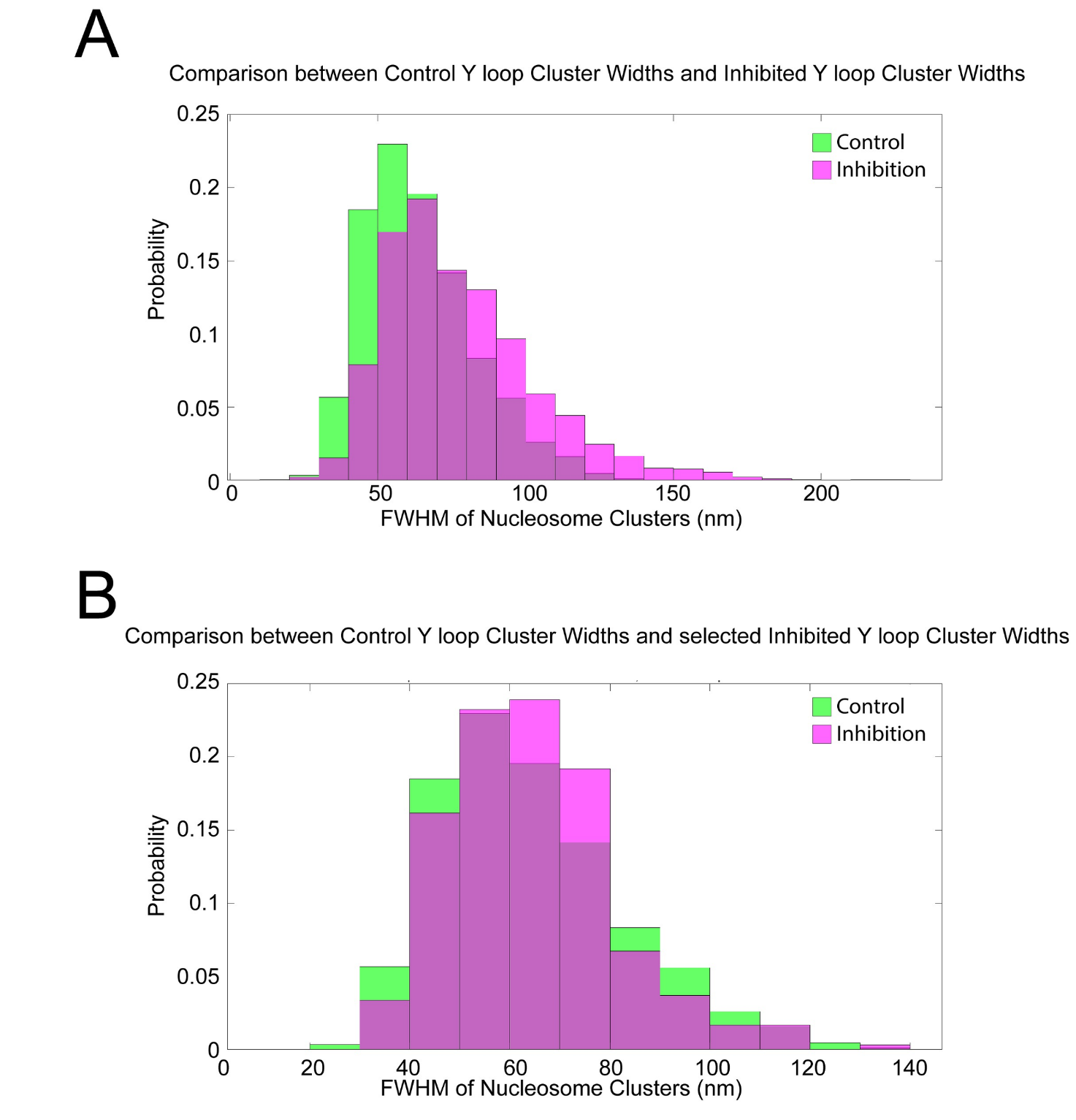


***Supplementary Figure 4.*** *(A) Overlapping histograms of the FWHMs of the control nucleosome clusters along the Y loops shown in green, with the FWHMs of the inhibited nucleosome clusters shown in magenta. The sample sizes are as in Suppl. Figure 3, as the same data is used. The frequencies were normalised and presented as a ‘probability’ value instead of raw frequency counts on the histogram for direct comparison between the two groups without influence of sample size differences. (B) Overlapping histograms of the FWHMs of the control nucleosome clusters along the Y loops shown in green, with the more selected FWHMs of the inhibited nucleosome clusters shown in magenta. Total cluster n = 297 for the finer selected data.*

The median FWHM for this data was 63 nm, more closely matching the median control FWHM Y loop chromatin cluster width. Both a two-sample Kolmogorov-Smirnov statistical test and Students T test accept the null hypothesis between these two datasets, demonstrating that the two distributions are not different to one another. We interpret this to indicate that the larger morphological changes that the Y loops undergo are separate from the mechanisms that generate the regular chromatin clusters.

***3. Quantification of association of RPol-PSer2 and –Pser5 with nascent RNA***

|  | PSer2 clusters | | | PSer5 clusters | | |
| --- | --- | --- | --- | --- | --- | --- |
| nucleus | total | +nRNA | ratio | total | +nRNA | ratio |
| 1 | 580 | 462 | 0.80 | 606 | 209 | 0.34 |
| 2 | 293 | 263 | 0.90 | 819 | 201 | 0.25 |
| 3 | 584 | 457 | 0.78 | 509 | 307 | 0.60 |
| 4 | 421 | 198 | 0.47 | 247 | 146 | 0.59 |
| 5 | 450 | 273 | 0.61 | 552 | 96 | 0.17 |
| 6 | 212 | 146 | 0.69 | 419 | 175 | 0.42 |
| 7 | 203 | 129 | 0.64 | 425 | 100 | 0.24 |
| 8 | 355 | 190 | 0.54 | 804 | 259 | 0.32 |
| 9 | 224 | 173 | 0.77 | 1010 | 377 | 0.37 |
| 10 | 311 | 251 | 0.81 | 551 | 248 | 0.45 |
| mean |  |  | **0.70** |  |  | **0.38** |
| stdev |  |  | 0.14 |  |  | 0.14 |
| p-value | **3.16E-05** |  |  |  |  |  |

***Supplementary Table.*** *Total number of nucleoplasmic RPol-PSer2 and –PSer5 clusters and of clusters associated with nRNA, their ratio for each measured nucleus, and the arithmetic mean, standard deviation and p-value for the mean ratios of the datasets.*

Differential association of RPol-PSer2 and –Pser5 with nRNA was measured by the ratio of the number of the respective RPol clusters touching nRNA accumulations over the total number of that RPol species in the nucleoplasm. 16-bit tiff stacks of single nuclei were cropped in Fiji from N2V3D denoised confocal stacks of whole-mount *Drosophila* testes stained for RPol-PSer2 or –Pser5 and for nascent RNA (nRNA; see main methods) and imported into Vision 4D software (Arivis). nRNA was segmented with the machine learning method on the EU channel, RPol with the “Blob Finder” by setting a diameter of 200 nm and a split sensitivity of 100 %, and the nucleoplasm with the “Draw Objects Tool”. The “Compartments” tool was used to isolate the nucleoplasmic fraction of RPol and to select RPol clusters touching nRNA by including objects inside and intersecting with a minimum overlap of 0 %. Segmentations were performed for each nucleus individually to compensate for differences in labelling and background. The parameters were optimized to correspond to the visual appearance. Statistics were performed with Excel (Microsoft). The p-value was calculated with the t.test function (1-tail, homoskedastic).
