# Supplementary figures and images for: The anatomy of transcriptionally active chromatin loops in Drosophila primary spermatocytes using super-resolution microscopy
Source: PLoS Genet. 2023 Mar 3;19(3):e1010654. doi: 10.1371/journal.pgen.1010654 (PMC10016678; doi:10.1371/journal.pgen.1010654)

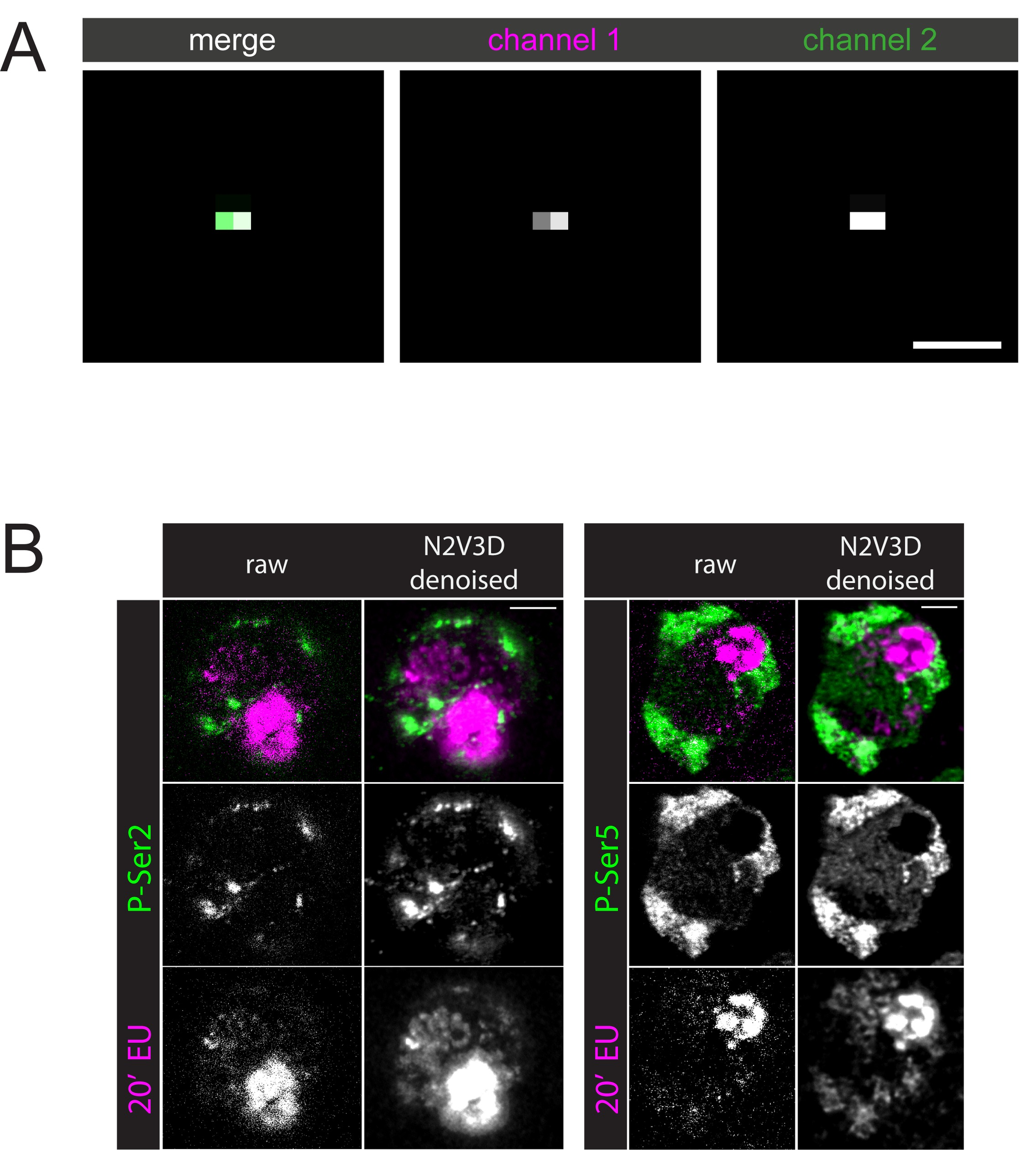

Supplement: S1 Fig — (TIF) [file pgen.1010654.s002.tif]
