# Supplementary material for: The anatomy of transcriptionally active chromatin loops in Drosophila primary spermatocytes using super-resolution microscopy
Source: PLoS Genet. 2023 Mar 3;19(3):e1010654. doi: 10.1371/journal.pgen.1010654 (PMC10016678; doi:10.1371/journal.pgen.1010654)
Supplement: Attachment — Submitted filename: Response_to_reviewers_revised.docx [file pgen.1010654.s005.docx]

In this revised response to reviewers we indicate the revised text in square brackets.

In this paper we exploit the model system of the Y loops in *Drosophila* spermatocytes to provide a super-resolution view of the topology of active transcription. We are pleased that Reviewer 1 assesses the work as “highly significant”. They also mention the caveat that findings may be “specific to the model system”. Reviewer 2 provides a counter to this caveat by pointing out that our findings are in line with some previous reports in mammalian cells. The interpretation in previous studies has however been hindered by the density of chromatin in the nucleus making it difficult to clearly identify individual fibres and Reviewer 2 points out that our work provides a significant advance as in our system we are able to “visualize the organization of chromatin within an individual, isolated loop”.

*Reviewer #1*

*∙ Have you assessed if you are under-labeling the nucleosomes? or RNA Polymerase?*

Yes, we tried a range of antibody concentrations which did not change the observations. We have added a statement in the M&M; “To control for under-labelling of structures, we tested a range of antibody concentrations and found no significant difference in the observed nucleosome or RNAPol distribution.”

*∙ Can you stimulate and inhibit transcription to see how changes in RNA Polymerase and nucleosome organisation occur?*

We are not aware of an approach to further stimulate transcription in this system, however we have some preliminary analyses of the effects of transcription inhibition that we plan to firm up within weeks. We agree with the reviewer that it may potentially be interesting and informative to include such studies but we do not consider this as central to the significance of the paper. So our plan is to attempt to quickly gather data on this for potential inclusion but we would plan to only include this material if we can rapidly achieve a clear understanding of the effects of transcription inhibition on nucleosome cluster organisation.

[In the revision, we include data from experiments where we have inhibited transcription (Fig 9 and text). We include validation of the inhibition in the Supplementary Information. We observe that, while transcription inhibition affects the overall morphology of the loops, it has little effect on the local nucleosome cluster organisation. This provides support to the view that the local clusters are formed independently of transcription. Quantification of clusters, in control and transcription inhibition conditions, is provided in the Supplementary Information.]

*∙ The 300 msec exposure time is very long for typical STORM experiments - blinking would usually be lost at this time scale?*

Apologies, this was a typo. Now corrected to 30ms.

*∙ Please quote laser power in absolute values rather than percentage*

Done.

*∙ Collecting variable number of frames (20K - 50K) can impact the number of detections/clusters. How can you account for this variation in your analysis?*

Although data was collected with variable number of frames (20K-50K), in the original paper we only used data from 50K samples for quantitative analysis. In response to reviewer #2, we have now performed the analysis on a larger data set with variable frame number. Analysis of the two data sets controls for any impact of frame number variation and makes very little difference to the results of the analysis e.g the median cluster FWHM remains at 52 nm.

*Reviewer #2*

*• Pages 2/3, the authors estimate the number of nucleosomes per cluster identified in the Y loops. They do so "based on a simple volume calculation" but the quantification method and reference values used to reach the estimate of a "maximum of 158 nucleosomes" is not reported in the methods. Also, the authors should discuss how do they account for multiple blinking effect in the estimate of cluster measures. Methods based on normalization curves with nucleosome arrays (Ricci et al Cell 2015) and on DNA origami (Cella Zanacchi et al Nat Meth 2017) among others were previously used to address similar questions. The authors should consider applying a normalization approach to achieve a more accurate estimate or at least discuss the caveats of their strategy.*

We have now specified the details of our estimation of the number of nucleosomes per cluster in the Materials and Methods. Techniques to quantify clusters based on localisation number have been used in the past on SMLM data, but the normalisations are difficult to validate as there is uncertainty around numbers of antibodies binding, differences in frame numbers affecting blink number, as well as unknown binding efficiencies of antibodies. Our cluster quantification instead is based on the size of the clusters (cluster width), as this does not vary significantly across frame number once signal saturation has been achieved and avoids the difficulties associated with normalisation of localisation number. As we have mentioned in the Discussion, our “simple volume calculation” does have some caveats arising from the STORM precision, the labelling method and the EM-derived estimate of packing density; nevertheless, as we say in the Discussion we consider it provides a useful “starting point for interpretation of the observed labelling in terms of underlying chromatin structure.”

*• Methods, STORM imaging: the authors should provide the imaging buffer composition as the papers they refer to make use of an array of buffers. If possible, the authors should include laser powers in standard units (kW/cm^2) to increase clarity and reproducibility.*

We have provided the imaging buffer composition and the laser power in standard units.

*• Methods, Cluster analysis: the authors should specify the values for the parameters used in "First a description of the average cluster was established using spatial statistics, then a clustering algorithm using the average parameters provided by the initial spatial statistics further refined the description"*

As detailed in response to the next comment, we have clarified the text in the Materials and Methods to specify the parameters and workflow of the analysis.

*• Given the availability of broadly used methods for clustering such as DBScan (Ester, et al. A density-based algorithm for discovering clusters in large spatial databases with noise. Kdd, 1996), Bayesian methods (Rubin-Delanchy et al. Bayesian cluster identification in single-molecule localization microscopy data. Nature methods, 2015), (Ricci et al. Cell 2015), Voronoi based tessellation (Levet et al. SR-Tesseler: a method to segment and quantify localization-based super-resolution microscopy data. Nature methods, 2015), and more recently machine learning approaches (Williamson et al. Machine learning for cluster analysis of localization microscopy data. Nature communications, 2020) the clustering method of choice is peculiar. It makes more difficult the direct comparisons with previous work. The authors could justify the choice or compare results using other more broadly used methods.*

We have added text to justify our selected analysis method and to clarify the analysis workflow. We do not consider our approach “peculiar” as very similar approaches have been employed previously (e.g. Sengupta et al Nat. Methods 8, 969–975 (2011), Veatch et al PLoS ONE 7, e31457 (2012) and Cisse Science 341:664-667 (2013)). In selecting this approach we compared various approaches and we found Meanshift to perform best with respect to identifying clusters closely positioned along a fibre. (Example data comparing the performance of Meanshift with DBscan in cluster identification with varying radius input parameter was provided to reviewers). We concluded that MeanShift provides a more robust cluster identification and that the radius value of 2Xsigma derived from the PCF is appropriate.

*• No statistical tests were performed in the study, although no comparisons between experimental conditions are shown. The authors mention the number of replicates performed only on Fig 3 ("13 regions of interest (ROI) were selected from 3 cells"), which is a low number of cells analyzed. The authors should enlarge the analyzed datasets and mention how many replicates were used throughout the work.*

As mentioned above, the quantitative analysis originally presented used the data with the constant, highest frame number (3 cells, 895 clusters). In the revised paper we present the analysis of the wider data set (12 cells, 2473 clusters). In practice this makes very little difference to the results, e.g the median cluster FWHM remains at 52 nm.

*• It is unclear why the authors did not perform STORM on EU-labeled samples immunolabeled with Pol2 antibodies. In principle, they could have applied the same imaging protocol used in previous figures gaining spatial resolution to better characterize Y nascent transcripts and how Pol2 is arranged with respect to nascent RNA.*

There are indeed a number of questions that can be approached by super-resolution imaging of nascent transcripts in association with co-labelling for RPol. We are exploring this interesting direction but this is a whole separate project and we propose to report our findings on this in a subsequent publication.

[We had further discussion with the editors on this point. The editors accepted our opinion that, for the question we were asking, confocal imaging is more appropriate than STORM. We have added text to indicate our reasoning; “This analysis was undertaken using confocal microscopy, as opposed to using STORM, as the confocal system is more suitable to provide the larger 3D depth needed to understand the distribution of nascent transcription along the Y loops that span almost the entire nucleoplasm”. At the editors’ suggestion we have added quantitative analysis (Supplementary Information) supporting our conclusions on the differential association of nascent RNA with Rpol-PSer2 and Rpol-PSer5.]

*• The text is well organized and clear as well as the figures but the authors should revise the text in the section "linking RNA polymerase distribution and Y loop transcription" to include missing references to Figure 10. For example, the statement "This distribution of nascent transcript along the Y loops fits with the distribution of RNA-PSer2," refers to results shown in Fig 10A.*

We are pleased the reviewer found the text and figures well organised and clear. We have added/modified figure references in this section to clarify the relationship between text and figures.

*• At end of page 5 the authors describe the location of RNA Pol2 pSer2 with respect to nucleosomes/RNA. As these results are related to findings reported by Castells-Garcia Nucl Acids Res 2022, cited elsewhere in the manuscript, the authors could cite the work here.*

Done.

*• It is unclear for which experiments goat anti-mouse Ig-Alexa Fluor 405 has been used, remove from antibody list if not relevant.*

This antibody has been removed.

*• The use of the 488 laser as the activatory laser is peculiar, can the authors better explain this choice?*

Empirically we found the 488 laser performed better as an activatory laser- as it gave a higher signal to noise than the more commonly used 405 laser. We have added this statement to the M&M.

[In addition, at the suggestion of the PLOS Genetics editors, we have clarified the meaning of the colours in Fig 3B.]
